# New insights into detecting alizarin from autofluorescence in marked glass eels

**DOI:** 10.1038/s41598-022-18440-0

**Published:** 2022-09-26

**Authors:** Mélanie Gaillard, Edith Parlanti, Mahaut Sourzac, Franck Couillaud, Coralie Genevois, Sébastien Boutry, Christian Rigaud, Françoise Daverat

**Affiliations:** 1 INRAE, UR EABX, 50 Avenue de Verdun, 33612 Cestas Cedex, France; 2 Univ. Bordeaux, CNRS, Bordeaux INP, EPOC, UMR 5805, 33600 Pessac, France; 3 Univ. Bordeaux IMOTION (Molecular Imaging and Innovative Therapies in Oncology), EA 7435, Case 117, 146 rue Léo Saignat, 33076 Bordeaux, France

**Keywords:** Marine biology, Ecology, Imaging, Microscopy, Optical spectroscopy

## Abstract

Alizarin detection in fish fins is extensively employed because it is easy to use. However, in eels, the eelGFP fluorescent protein may impede the detection of the fluorescent markers in the eel tissues. The study tests the effectiveness of three of the most up-to-date alizarin-detecting technologies on the living body and fins of European glass eels (*Anguilla anguilla* L.). The findings demonstrated that the control group had a high autofluorescence at alizarin and eelGFP maxima bands. With fluorescence reflectance imaging (FRI), the eel living body autofluorescence impeded the detection of the marked eels. In contrast with experimental excitation-emission-matrix (EEM) fluorescence analyses, 99% of the marked eels were correctly assigned to their group from fluorescence analyses of their fin cellular contents. With epifluorometry (EPI), 100% of the marked eels were detected with the caudal fin tips when excited at 450–490 nm wavelengths due to a weaker autofluorescence signal. EEM and FRI assays unveiled an average fluorescence quenching 60% and 44% of the marked group respectively, in the alizarin and eelGFP maxima bands. The fluorescence quenching observed is discussed. Results will benefit experimental design by examining autofluorescence effects on mark detection and the development of non-invasive detection methods in this critically endangered species.

## Introduction

Studies have extensively demonstrated how to detect fluorescent stains in the fins of marked fish (guppie^1^; zebrafish^2^; trout^3^; tilapia^4^; sturgeon^5^). In eels, the fin tips are widely used in genomics studies^6^ and recently, in stable isotope studies^7^. However, eel fins are not yet employed in alizarin mark detection studies.

Recently, a novel fluorescent antioxidant protein was discovered from the eel’s skeletal muscle, named “eelGFP”^8^ or “UnaG”^9^. This eelGFP produces green fluorescence when binding with bilirubin. EelGFP emits a major fluorescence peak at 493 nm excitation wavelength and at 527 nm emission wavelength^8,9^. EelGFP shows another minor absorption peak at 280 nm and the absorption ratio 280/493 nm is 0.08^9^. The fluorescence of eelGFP may impede alizarin mark detection in the eel tissues as alizarin and eelGFP emit fluorescence at close-wavelengths under green light sources.

In European eels (*Anguilla anguilla* L.), alizarin marking is the method used to monitor marked eels that have been transferred for restocking purposes. As an endangered species that have faced dramatical population collapse since the eighties^10^, eel management programs were established in 2007 in an effort to protect and restore the species. One conservation measure involves reserving 60% of glass eel catches for restocking, and in France, 30% of which are alizarin mass-marked before their transfer^11^. Restocking eels consists of transferring juveniles caught in coastal or estuarine habitats into freshwater habitats^11^. Thus each year in Europe, millions of glass eels were mass-marked with alizarin red S (at 150 ppm) or alizarin complexone (at 50 ppm) mostly for marking eel otoliths^12–14^. However, detecting the recaptured marked eels by analysing their otolith is a lethal method.

The present study aimed to test three new methods for detecting alizarin-marked glass eels on body parts other than otoliths. Three up-to-date technologies were used: (i) fluorescence reflectance imaging (FRI) on the living body of anaesthetized glass eels; (ii) fluorescence excitation-emission matrices (EEM) of the caudal fins cellular content and (iii) epifluorescence microscopy (EPI) on caudal and pectoral fin tips.

## Results

### Fluorescence reflectance imaging (FRI)

FRI provides measurements of fluorescence in radiance (p/sec/cm^2^/sr). Accordingly to the fluorescence of a drop of alizarin solution (red S; 150 ppm), FRI analyses were performed with two sets of excitation and emission wavelength filter: (1) 450–480 nm at excitation, 515–575 nm at emission; (2) 485–515 nm at excitation, 515–575 nm at emission.

**Figure 1:**
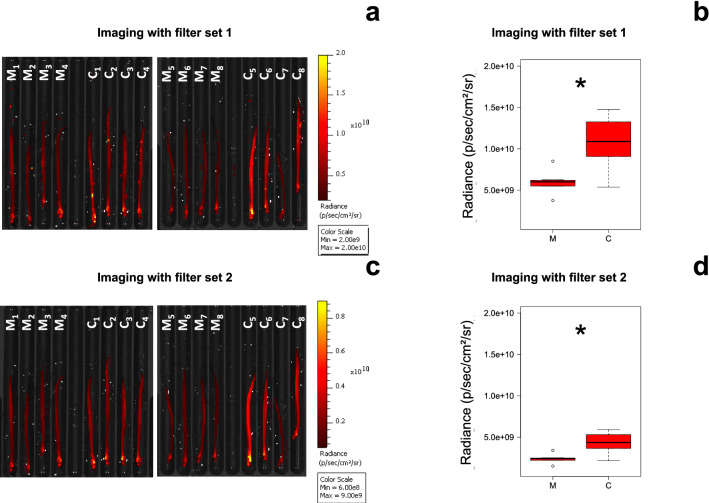
In vivo fluorescence reflectance imaging (FRI) of glass eels. Radiance of marked and control glass eels measured by FRI: (**a**) imaging and (**b**) boxplot with the filter set 1 at 450 nm–480 nm_515 nm–575 nm Ex_Em wavelengths; (**c**) imaging and (**d**) boxplot with the filter set 2 at 485 nm–515 nm_515 nm–575 nm Ex_Em wavelengths. Radiance is the fluorescence intensity expressed in p/sec/cm^2^/sr (photon/second/cm^2^/steradian). M: marked group. C = control group. *significant differences.

The radiance of the live glass eels were significantly different between the two filter sets (W = 18.6; *p*-value < 0.00001, Fig. 1). The radiance of the live glass eels was significantly twice as high with filter set 1 (Fig. 1a,b) than with filter set 2 (Fig. 1c,d). However, the quality of the imaging with filter set 2 was better as there was less background noise.

The radiance between groups was significantly different (W = 4.3, *p*-value = 0.038). The radiance of the control group was significantly higher than the radiance of the marked group for any filter set. With the filter set 1, the radiance of the control group averaged 1.12 ± 0.30 × 10^10^ while the marked group averaged 0.64 ± 0.17 × 10^10^ (Fig. 1b, W = 5.6, *p*-value = 0.0179). With the filter set 2, the radiance of the control group averaged 0.45 ± 0.12 × 10^10^ while the marked group averaged 0.25 ± 0.07 × 10^10^ (Fig. 1d, W = 54, *p*-value = 0.0238).

The fluorescence of the marked glass eels was significantly lower compared to the control group paricularly when looked at the head of the control glass eels named C1, C2, C3, C5, C6 and C8 that peaked at 2 × 10^10^ radiance with filter set 1 (Fig. 1a) and at 0.84 × 10^10^ radiance with filter set 2 (Fig. 1c). This difference in radiance bewteen the head and the body for the marked (W = 1.8, *p*-value > 0.05) and the control group (W = 3, *p*-value > 0.05) was not significant.

Although the radiance of alizarin was, for example with filter set 2, of about 1.8 to 2 × 10^8^ (Supplementary Fig. S2b), alizarin did not increase quantitatively the radiance measured in the marked group. In contrast, a higher fluorescence was observed in the control group. Moreover, the fluorescence quenching percentage ratio Q (%) of the marked group was about 44% regardless of the filter set fixed for the imaging.

### Excitation-emission matrix (EEM) fluorescence spectroscopy

**Figure 2:**
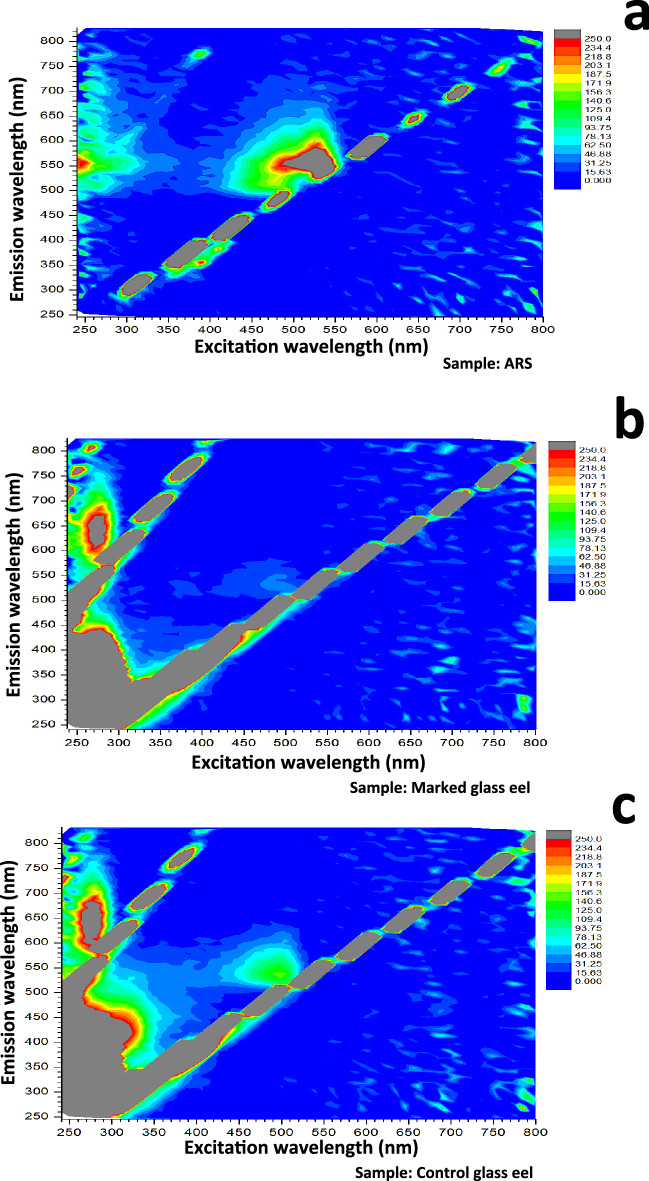
Excitation-Emission Matrix (EEM) fluorescence spectra. The EEM spectrum of (**a**) an alizarin red S solution (150 ppm) and as examples of EEM spectra obtained the EEM spectrum of (**b**) a marked glass eel and (**c**) a control glass eel. Fluorescence intensities are given in arbitrary units (a. u.).

The UV–vis absorption spectrum of an alizarin solution (alizarin red S, 150 ppm) showed three peaks of absorption: (1) at 240–280 nm, (2) 310–360 nm and (3) 400–550 nm wavelengths (Supplementary Fig. S3). The EEM spectrum of alizarin showed two fluorescence peaks in accordance with its absorption spectrum (Fig. 2a): (1) one peak of up to 234 arbitrary units (a. u.) between the wavelenghts 240–360 nm at excitation and 500–600 nm at emission and (2) the greatest peak of more than 250 a. u. between the wavelengths 400–550 nm at excitation and 480–650 nm at emission. The alizarin fluorescence was maximal between the wavelengths 490–550 nm at excitation and 545–570 nm at emission (Fig. 2a).

**Figure 3:**
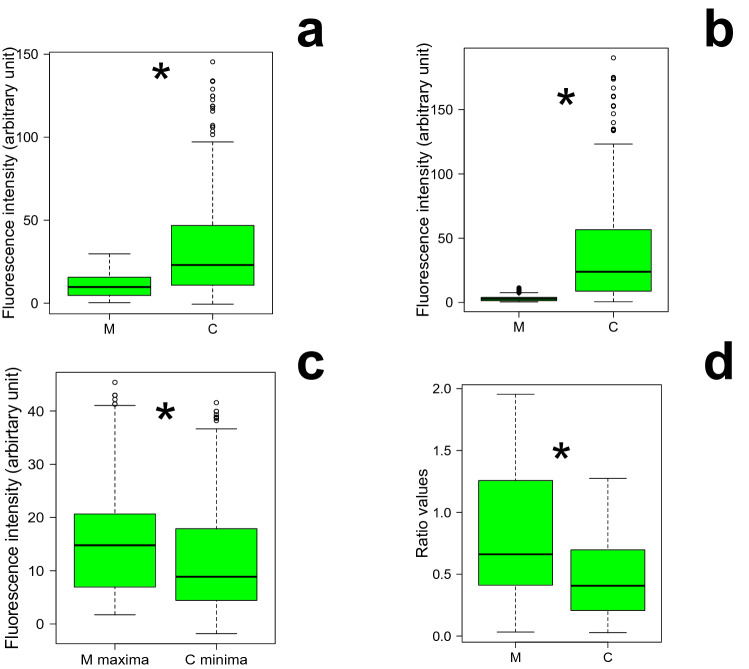
Boxplots of the fluorescence intensities of the EEM spectra of marked and control glass eels and their values of the selected fluorescence ratios. Boxplot of the fluorescence intensities (**a**) means, (**b**) standard deviations (**c**) maxima and minima measured whithin the bands of the alizarin peak (2) and (**d**) boxplot of the fluorescence ratio values that have been selected. M: marked group (n = 12). C: control group (n = 12). *: significant differences.

**Figure 4:**
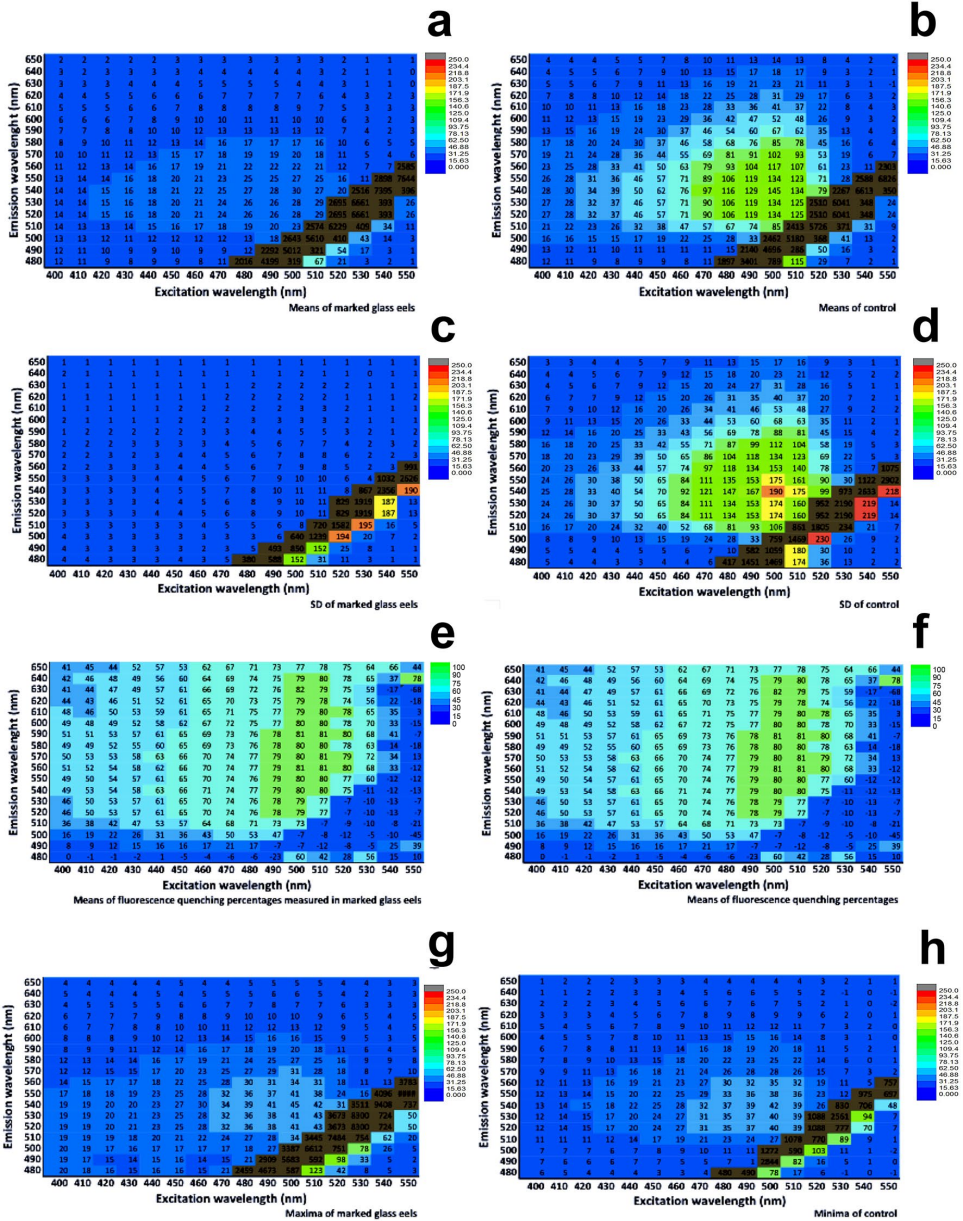
Excitation-Emission Matrix (EEM) spectra of glass eels in the alizarin peak. EEM of fluorescence intensities means of (**a**) marked and (**b**) control glass eels; EEM of fluorescence intensities standard deviations of (**c**) marked and (**d**) control glass eels; EEM of (**e**) means and (**f**) standard deviations of the fluorescence quenching percentage measured in the marked group; EEM of the fluorescence intensities (**g**) maximum of the marked glass eels and (**h**) minimum of the control glass eels. Fluorescence intensities are in arbitrary units (a. u.) and presented for each excitation-emission wavelength (numbers in the cells). n = 12 in each group.

The EEM spectra of the control and marked group showed maximum fluorescence at the same wavelengths as alizarin and at other wavelengths corresponding to the natural signal of the fish: between 240 and 360 nm at excitation (Ex) and between 250 and 450 nm at emission (Em). At the peak of alizarin (2): 400–550 nm_480–650 nm Ex_Em wavelengths, the fluorescence of the marked group was consistently lower than for the control group. This difference in fluorescence intensity between the groups was observable on EEM spectra (see as examples of EEM spectra obtained, an EEM spectrum of a marked and of a control glass eel in Fig. 2b,c). Thus, when the EEM spectra of glass eels were enlarged in this alizarin peak (2), the mean fluorescence intensities of the marked group were significantly lower than the control group (W = 46,360, *p*-value < 0.00001; Fig. 3a). The fluorescence of the marked group was from 6 to 25 arbitrary units, a. u. (Figs. 3a, 4a). By contrast, the control group emitted a brighter fluorescence from 13 to 100 a.u. about 40 to 80% higher than the marked group (Figs. 3a, 4b). Moreover, the fluorescence intensities standard deviations differences between the groups were highly significant (W = 56,296, *p*-value < 0.00001; Fig. 3b). The fluorescence variation of the marked eels were weak and close to 1, from 0 to 11 a.u. (Figs. 3b, 4c) while the control group emitted a variable fluorescence from 10 to 190 a.u. (Figs. 3b, 4d).

The EEM spectra analyses revealed how the fluorescence of the marked group is weak and how autofluorescence in the control group is variable and high in the alizarin peak (2): 400–550 nm_480–650 nm Ex_Em wavelengths. The percentage of fluorescence intensity quenching Q (%) in marked eels was evaluated between these wavelengths. Q-levels were high in marked eels (Fig. 4e) and were maximum of about 80% at wavelengths between which the alizarin fluorescence was maximum: between 490–530 nm_545–570 nm Ex_Em wavelengths. The standard deviations of Q (%) were variable (Fig. 4f) with respect to the fluorescence variability of the control group (Fig. 4d). The values of Q (%) demonstrated a fluorescence quenching in the marked group at these wavelengths. However, whithin the natural fish signal bands between 240–360 nm_250–450 nm Ex_Em wavelengths, the difference in the fluorescence intensity between both groups was not significant (W = 29, *p*-value > 0.05; Fig. 2b,c). Their fluorescence intensity at these wavelengths was high about 3277 a.u. These results suggested that the fluorescence quenching of the marked group measured in the alizarin peak (2) would be linked to the eel autofluorescence and the alizarin fluorescence.

To test whether this fluorescence quenching in marked eels may be a detection-tool for this group, their maximum fluorescence intensities should be lower to the minimum fluorescence intensities of the control group (Figs. 3c, 4g,h). However, the maximum fluorescence intensities of the marked eels were significantly higher than the minimum fluorescence intensities of the control eels (W = 37,632, *p*-value < 0.00001). The maximum and the minimum fluorescence values of the groups overlapped (Fig. 3c). Thus, no threshold value of maximum fluorescence of the marked group could be defined and their maximum fluorescence intensities cannot be used as a detection tool for marked eels (Fig. 4g,h).

QDA were run with the first, second and three quartiles of fluorescence intensities of the control and marked groups. The percentage of correct classification of the 468 observations was the highest with these three variables (Table 1). The percentage of correct assignment to a group of QDA models run with the third quartile was indeed 61%, with the third and second quartiles, 78% and with the three quartiles, 82%. Almost all the marked eels (99%) were assigned to their group with QDA model run with the three quartiles. This model discriminated the fluorescence intensities of the marked group, comprised between 4.68 and 15.59 a. u. quartile 1 and quartile 3 respectively, and those of the control group, comprised between 10.82 a. u. and 46.83 a. u. (Table 1).

**Table 1:** Results from quadratic nonlinear discriminant analysis (QDA).

	QDA Model correctness rate	Marked	Control
Fluorescence intensities (n = 468) from EEM measured between 400–550/480–650 nm excitation/emission wavelengths	Q1 + Q2 + Q3: 0.8205	99.14% (n = 232)	64.53% (n = 151)
Quartile 1 (Q1) Quartile 2 (Q2) Quartile 3 (Q3)		4.68 9.72 15.59	10.82 23.00 46.83
Values (n = 38) of the 19 ratios	With the 19 Ratios: 0.6053	57.90% (n = 11)	63.16% (n = 12)
Means ± SD		0.79 ± 0.50	0.49 ± 0.36

QDA to assign to a glass eel group EEM fluorescence intensities measured in the alizarin maximum bands and values of the ratios R. The model correctness rate, the percentages of each assignation (with the number of observations in parentheses), the fluorescence intensities quartiles and the ratios values means ± SD are presented for each group.

**Figure 5:**
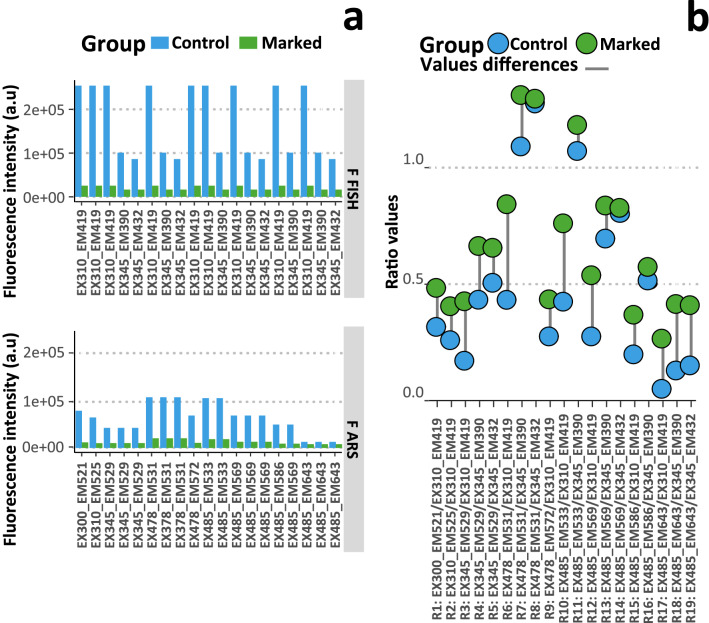
Fluorescence peaks and their ratios. (**a**) Graph plot of the fluorescence intensities of peaks within the alizarin signal (F_ARS_) and within the natural fish signal (F_FISH_) used in the alizarin fluorescence ratios (R_n_). (**b**) Graph of the values of the 19-alizarin fluorescence ratios (R_n n:1→19_) that discriminated the marked (n = 12) from the control (n = 12) glass eels. The grey lines represent the differences in the ratios values between the groups. R is the ratio of F_ARS_ to F_FISH_.

Another approach for detecting alizarin in marked fish was to compare between marked and control groups, rates of fluorescence intensities ratios (R) in the alizarin signal (F_ARS_) to those in the natural fish signal (F_FISH_) (Fig. 5). When the rates were higher for the marked group than for the control group, the R was selected. The intensities of the F_ARS_ and F_FISH_ selected are presented for each Ex_Em wavelengths (Fig. 5a). The F_ARS_ were chosen at the edge of alizarin peak to measure as less autofluorescence as possibe in order to better detect alizarin such as intensities observed at 485 nm_643 nm Ex_Em wavelengths, the F_ARS_ of R_18_ (Fig. 5a). The marked group had significantly higher fluorescence R-values with 19 R (R_1_ to R_19_) (W = 279, *p*-value = 0.01098; Fig. 3d). The average difference of the R-values between marked and control groups was 18% (0.18 ± 0.10, Fig. 5b). Thus, the fluorescence of marked glass eels was on average 18% higher than the control glass eels and the highest of 41% with R_6_ (*F*_*Ex478_Em531*_/*F*_*Ex310_Em419*_), 33% with R_10_ (*F*_*Ex485_Em533*_/*F*_*Ex310_Em419*_), 29% with R_18_ (*F*_*Ex485_Em643*_/*F*_*Ex345_Em390*_) and 26% with R_19_ (*F*_*Ex485_Em643*_/*F*_*Ex345_Em432*_), R_12_ (*F*_*Ex485_Em569*_/*F*_*Ex310_Em419*_) and R_3_ (*F*_*Ex345_Em529*_/*F*_*Ex310_Em419*_ ; Fig. 5b). The 38 R-values were used in a QDA to discriminate the groups (Table 1). The mean percent correct assignation of the model was 61%. The marked glass eels were assigned to 57.60% in their group and the control glass eels were assigned to 63% in their group. The model was able to classify one glass eel out of two.

### Epifluorescence microscopy (EPI)

**Figure 6:**
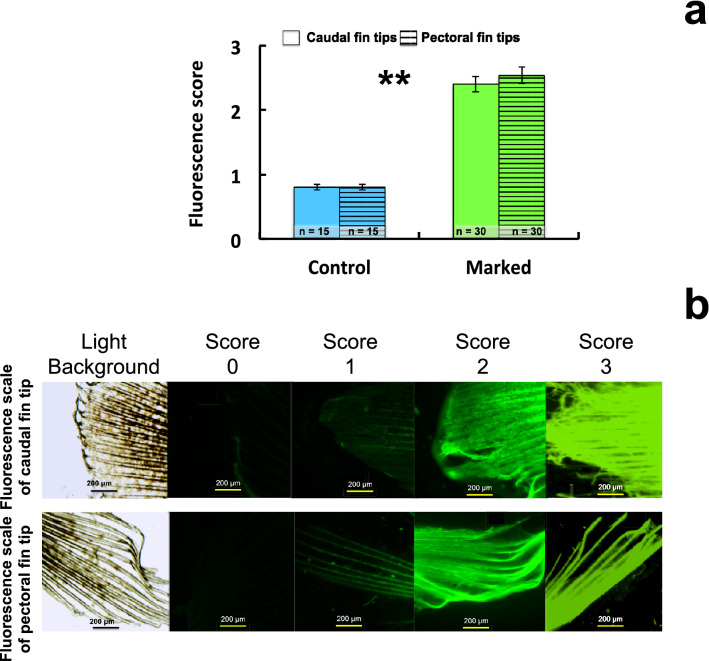
Epifluorescence scores of caudal and pectoral fins of marked and control glass eels. (**a**) Means of the epifluorescence scores assigned to caudal and pectoral fin tips of marked and control glass eels. *: significant difference between marked and control glass eels for a type of fin. (**b**) Epifluorescence scores scale 0–3 (0, no fluorescence; 1, weak autofluorescence around fin rays and at the edge of the fin; 2, bright and heterogeneous fluorescence of alizarin stain on fin rays and tissue; 3, very bright and homogeneous fluorescence of alizarin stain on fin tip).

The fluorescence scores assigned to the control group (n = 30) were not significantly different between caudal and pectoral fins (χ^2^ = 2.109; df = 4; *p*-value > 0.05; Fig. 6a). The control glass eels had a majority score of 1 except for three individuals. Thus, in the control group, 20% of the pectoral and caudal fins did not fluoresce and 80% of them emitted a low intensity of autofluorescence. There was no variability between the research operators for assigning a score to this group.

The fluorescence scores assigned to the marked group (n = 60) were not significantly different between caudal and pectoral fins (χ^2^ = 9.008; df = 4; *p*-value > 0.05; Fig. 6a). The fluorescence assigned to this group was brighter and more intense compared to the control group. The majority of marked glass eels had a score between 2 and 3. More precisely, scores assigned to caudal fins were 2 for 67% of them and 3 for 33% of them. By contrast, scores assigned to pectoral fins were 3 for 60% of them, 2 for 33% and 1, a score that can be confused with a control, for 7% of them. The variability in assigning a score (± 1) between the three researchers was low: 11% for caudal fins (n = 5/30) and 18% for pectoral fins (n = 7/30).

The difference in fluorescence scores between groups was highly significant for both caudal (χ^2^ = 44; df = 4; *p*-value < 0.00001) and pectoral fins (χ^2^ = 36,457; df = 4; *p*-value < 0.00001). 93% and 100% of marked glass eels were detected by analysing the pectoral and the caudal fin fluorescence respectively. The results showed significantly that the alizarin-marked glass eels emitted greater and brighter fluorescence than the control group for both fins. The difference in fluorescence between the groups was highly significant (Table 2).

**Table 2:** The 15 fluorescence peaks analysed in the glass eels with their excitation and emission wavelengths.

Excitation wavelengths (nm)	Number of peaks	Emission wavelengths (nm)	
		Within the ARS maxima band	Within the fish autofluorescence signal
300	3		332
			400
		521	
310	3		345
			419
		525	
345	3		390
			432
		529	
478	2	531	
		572	
485	4	533	
		569	
		586	
		643	

## Discussion

Alizarin detection in marked eels and autofluorescence in eels were examined over a wide wavelength spectrum using different modern optical imaging technologies. Two alizarin peaks were detected by EEM analyses within these spectral bands: (1): 240–360 nm_500–600 nm excitation_emission wavelengths (2): 400–550 nm_480–650 nm excitation_emission wavelengths. These two alizarin peaks detected corroborate and explain the use of several excitation and emission filters to detect alizarin stains in the literature (Table 3).

**Table 3:** Studies of interest that analysed alizarin red S stains other than on otoliths.

Reference	Species	Material	Sample used	ARS light source	ARS excitation filter	ARS barrier or emission filter
Present study	European eel *Anguilla anguilla*	Fluorescence reflectance imaging (Lumina LT)	Living whole-body	In the Blue In the Green	450–480 nm 485–515 nm	515–575 nm
		Epifluorescence binocular (SMZ25, Nikon) with a Nikon B-2A fluorescence filter	Fin rays	Blue light	450–490 nm	510 nm 515 nm
		Fluorescence spectroscopy with an Aqualog spectrofluorometer (Jobin Yvon)	Cellular content of fins	From UV to IR	385–560 nm	480–670 nm
Liu et al. 2009	Japanese flounder *Paralichthys olivaceus*	Epifluorescence microscope (OLYMPUS BX51) with an Olympus DP70 high resolution digital camera	Otoliths, fin rays and scales	UV Blue light Green light	365 nm 490 nm 545 nm	420 nm 515 nm 590 nm
Bensimon-Brito et al. 2016	Zebrafish *Danio rerio*	Stereo microscope (Leica MZ36) with a F-View II camera	Skeletal tissue imaging	Blue light Green light	450––480 nm 510–550 nm	
Caraguel et al. 2015	European eel *Anguilla anguilla*	Epifluorescence microscope (OLYMPUS BX 51)	Otoliths	Green light	530–550 nm	590 nm
Bashey 2004	Guppies *Poecilia reticulata*	Epifluorescence microscope	Fin rays	Green light	545 nm	580 nm
Crook and O’Mahony 2009	Golden perch *Macquaria ambigua*	Stereomicroscope (MZ16 F Leica) with digital camera	Head, anal fin, caudal fin and otoliths	Green light	560–600 nm	610 nm
Ibanez et al. 2013	Nile Tilapia *Oreochromis niloticus*	Epifluorescence microscope (Zeiss Axio Start)	Otoliths, scales and caudal fin rays	Green light	560–600 nm	630–705 nm

Literature reference, species names, material, sample used, light source and filter used to detect alizarin (ARS) are presented for each study.

Our study have newly demonstrated that epifluorimetry significantly detected marked glass eels by analysing their fins. The blue excitation light source (450–490 nm excitation wavelengths) was effective in detecting marked from control glass eels. Our results are in accordance with^15^ who demonstrated that the blue light source provided a better detection of alizarin stain on fins in fish due to a higher stain intensity than with other light sources. In contrast, the filter set 1 fixed at blue excitation wavelengths in FRI analyses (450–480 nm excitation wavelengths) did not detect alizarin stains by imaging the living marked eel body. No such difference was observed in the EEM spectra and Q (%) was high (490–530 nm) in both blue and green excitation wavelengths. In both EPI and FRI methods, the excitation wavelengths were fixed below the excitation maximum (493 nm) of the eelGFP protein^8,9^. However, the discrepancy between EPI and FRI could be explained. In EPI, the eelGFP protein and eel autofluorescence was less excited in the fin tips because this tissue is thin, less muscle, less vascularised and the fin rays are near the skin surface. In fact, on the images from FRI, the tip of caudal fin is less observable corrobarating a lesser autofluorescence in this body part. Moreover, in FRI analyses the entire living body including other biological forms were imaged increasing the detection of autofluroescence. In EEM fluorescence spectroscopy, the cell material was extracted from the fin thus, high autofluorescence was detected from biological forms as observed in FRI analyses. In the EEM spectra, Q (%) was high (490–530 nm) in both blue and green excitation wavelengths. EEM spectra of control glass eels showed indeed how eels emitted autofluorescence at a large spectral band including the wavelengths of the fluorescence maximum of alizarin and eelGFP^9^. Although the emission wavelengths set in EPI (510–515 nm) and in FRI (515–575 nm) were at the edge of the eel autofluorescence peak (470–520 nm), only those set in EPI were below the eelGFP fluorescence peak (527 nm)^9^. Moreover in regard with our results, FRI bandpass although set to alizarin maximum fluorescence, was also set to the maximum fluorescence of eelGFP and other biological forms. Thus, our results strongly suggested that eel autofluorescence and eelGFP fluorescence was few excited in EPI analyses and consequently, alizarin fluorescence could be detected in eel tissues.

In EPI analyses, the fluorescence intensity of the marked group was brighter on pectoral fins than on caudal fins. However, all marked glass eels without exception were detected using caudal fins compared to the pectoral fins. Furthermore, the sampling of pectoral fin tips required the use of a binocular magnifier while the sampling of a caudal fin tips could be done without and is easier. While extracting the pectoral fins can affect the growth, stability, propulsion and the survival of the fish^16,17^ it is possible to extract a caudal fin clip without affecting the fish survival. Numerous studies have developed non-lethal detection method with caudal fin clips for small fish (> 50 mm)^1–4,6,18^ and already in European eels^7^. The caudal can indeed regenerate rapidly in fish and the caudal fin clip results in low mortality in early stages^2,4,7,17–21^. These results are promising for developing a non-lethal alizarin detection method in marked eels using caudal fins^7^.

This study is the first to examine the detection of fluorescent markers in fish using EEM fluorescence spectroscopy analyses. EEM fluorescence assays provided some advantages in the detection of alizarin from a piece of tissue. Firstly, a spectrofluorometer can detect alizarin at a low concentration and at trace levels in a homogenate (150 or 50 ppm in marking restocking protocol). Secondly, the homogenate of each caudal fin was easy to prepare and did not take much time. Moreover, the ultrasonic immersion of the fin suspended in the water (sonication) enables the releasing of the fluorescent dye from the tissues and the body structures of the fish^3^. Furthermore, most of the cellular content released by sonication was filtered (0.70 µm) prior to the analyses. Thus, another advantage using this new technique is that the fluorescence analysis is not impeded by the width of the tissues. In addition, the operating precautions for fluorescence (absorbance < 0.1) ensure linearity between fluorescence intensity and fluorophore concentration. Finally, the most interesting advantage is that only a little clip of caudal fin (5 mm) is needed to perform the EEM analyses, which are rapid and inexpensive.

Two approaches in EEM analyses were tested to detect alizarin in the caudal fins: the comparison between marked and control glass eels of (i) their maximum and minimum fluorescence intensities of their EEM spectra and of (ii) their rates for ratios between fluorescence peaks. The first approach showed how the fluorescence intensities of the marked group was lower than the control group. Thus, our results strongly support the hypothesis of a fluorescence quenching in marked eels linked to an effect between alizarin fluorescence and eel autofluorescence including eelGFP. As a matter of fact, the fluorescence of both groups was not different when their fluorescence was measured in the natural fish signal. By contrast, the marked eels fluorescence was lower in the maxima bands of alizarin and eelGFP. A fluorescence quenching to more 80% was measured in these bands. However, no threshold value for maximum fluorescence of marked glass eels have been determined. There was little overlap of the maximum and minimum fluorescence values between groups of about order 1 (a.u.). Perhaps a finer filtration, below 0.70 μm, of the extracted caudal fin prior to EEM analyses would have provided for more accurate detection of thresholds between groups. A longer sonication time was tested on some samples and did not improve the fluorescence signals. Nevertheless, the low fluorescence intensity values of marked glass eels enable to detect 99% of the marked group using a quadratic nonlinear model, the classification error of the model was 18%. These findings are very promising for detecting marked eels using the fluorescence quenching of marked eels and with 5 mm of caudal tissue.

In the second approach, different R and spectral regions were tested to detect alizarin in marked fish. The most promising R's for the detection of alizarin-marked fish were R_6_ (*F*_*Ex478_Em531*_/*F*_*Ex310_Em419*_), R_10_ (*F*_*Ex485_Em533*_/*F*_*Ex310_Em419*_) and R_18_ (*F*_*Ex485_Em643*_/*F*_*Ex345_Em390*_) for which the marked group emitted a higher fluorescence at 41%, 33% and 29% respectively. R_6_ appeared to be the most discriminating for detecting groups. However, the assignation of the groups with the ratio values was correct for 1 out of 2 individuals in a QDA model. The number of observations used in the model (n = 38) was maybe not enough for a higher assignation, it would be interesting to repeat the assays on a larger number of replicates. These first experimental analyses by spectroscopy and EEM enabled the development of new strategies to detect a fluorescent marker in the tissues of a fish caudal fin tip (5 mm). These experiments may lead to new approaches to develop non-lethal detection of marked fish. Our results show the relevance of analysing the full spectra of fish and alizarin fluorescent dyes to better understand the spectral behaviours of markers in fish.

Finally, this study demonstrated for the first time that alizarin and eel autofluorescence emit fluorescence at equivalent wavelengths whatever the optical imaging technology used. The literature showed that fish autofluorescence is mainly due to the fluorescence from blood proteins such as flavin, elastin and collagen^1,4,5,22,23^ and recently in eel, to protein eelGFB that fluoresces at close wavelengths of alizarin^8,9^. In this study, a fluorescence quenching of 44% with FRI and 65% with EEM assays was observed in the alizarin maximum fluorescence bands. Moreover, eel autofluorescence was not different between groups in the natural fish signal (240–360 nm_250–450 nm Ex_Em wavelengths). The EEM results strongly suggest that eel autofluorescence in the alizarin fluorescence band and eelGFP fluorescence could lead to a fluorescence extinction upon contact with alizarin marker. Fluorescence quenching can occur between two fluorescent molecules and diminish the time and fluorescence yield of fluorescent proteins in contact with inhibitors. Fluorescence quenching could be dynamic or static, resulting from the collision or creation of a temperature-dependent complex between the dye and the quencher^24^. To date, there are no references on fluorescence quenching by the eelGFB protein and its impact on eel biology. However, a few studies document a strong fluorescence quenching between alizarin and the liver protein albumin^24,25^ with which eelGFP interacts. Studies have already developed rapid detection methods for albumin based on the quenching effect of alizarin^26,27^. The fluorescence quenching in marked glass eels could be due to several causes. The marked glass eels may have secreted lower amounts of the antioxidant protein eelGFP during the oxygenated mass-marking bath, which may have contributed better to the binding of alizarin on eel tissues without interacting with the eelGFP autofluorescence. Unless the presence of alizarin did not allow eelGFP by competitiveness to bind to bilirubin or albumin^24^ and remained non-fluorescent during the mass-marking. The eelGFP fluorescence has indeed been shown to be enhanced when its activity is coupled with bilirubin and albumin^8^. Unless, the fluorescence quenching in marked glass eels is the result of two fluorescence quenching between alizarin-albumin^24–26^ and eelGFP-bilirubin. However, our results showed that the fluorescence of marked glass eels was very stable compared to the control group and may be the result of a biochemical effect. Further investigations are required to understand (a) the interactions between alizarin, eelGFP, bilirubin and albumin or other biologic forms and (b) the fluorescence quenching of the marked glass eels specifically in the alizarin and eelGFP maxima bands. In view of^27,28^, fluorescence quenching could be used to detect marked glass eels and be tested to develop a new approach for their detection.

Our results suggested some perspectives. The main limitation experienced in detecting fluorescent markers in eel tissues was detecting the marker in wavelengths outside of the eelGFP and eel autofluorescence peaks. Eel autofluorescence is a constraint to the use of fluorescent dyes such as alizarin red S. However, alizarin red S can be detected on fish fins after eight months^4^. This may also limit the use of alizarin for the eel long-term monitoring unless an additional external mark is done when the fins are clipped. Supplementary investigations are also needed to verify the viability beyond 8-months of the fin mark. Other authors preferred the use of alizarin complexone that was more effective than alizarin red S and our methods could be tested with^15^. The present study proposes new low-cost approaches that could be improved to address these limitations: EEM and EPI could be tested on older marked eels and FRI analyses should be potentially improved by using a narrower band pass that targets alizarin, specifically. Finally, the use of more expensive fluorescent dyes like the ones widely used in medical imaging such as cyanine 5 (Em: 670 nm), cyanine 7 (Em: 767 nm), infrared fluorescent protein (Em: 690 nm)^29,30^, would solve these limitations because they emit fluorescence in wavelengths that fall outside the scope of those present in biological forms. Conclusively, eelGFP offers new directions in marking and detection protocols.

To conclude, the results highlighted the relevance of analysing fish autofluorescence on a large spectrum of excitation and the emission wavelengths in detecting alizarin fluorescence from fish fins. Further research is needed to test how to take advantage of the fluorescence quenching in the detection of marked eels. These results will benefit the development of new non-lethal detection methods and the knowledge of eel autofluorescence and its biological role in individuals.

## Methods

### Glass eels collection (*Anguilla anguilla,* L.)

Three hundred European glass eels (< 7 cm) were collected in two French rivers, at Saujon in July 2016, at Pas de Bouc in June 2018 and in the Gironde Estuary in February 2018 and 2019 (Supplementary Fig. S1). A Hundred of glass eels from the Gironde Estuary were marked by Fish-Pass in a bath of 150 ppm of alizarin red S for 3 h (see details of the marking protocol in^12^). Before being sampled, especially the marked and control glass eels from the Gironde estuary were transferred at INRAE Saint-Seurin experimental station (animal experimentation approval number A33-478-001) for one month of rearing. After two hours of acclimation, these glass eels were divided equally into 4 baskets of control or marked glass eels set into an outdoor 14 m^3^-protected pool, continuously aerated and filled with well water. In each basket, the mean density was 40 g m^−2^. These young eels were fed ad libitum*.* After one month, alive young eels were collected for FRI analyses. In total, the analyses were conducted on 85 individuals with control (n = 35) and marked (n = 50) glass eels.

### 2D Fluorescence reflectance imaging (FRI)

In vivo FRI is an innovative and non-intrusive technique for fluorescence detection on live animals. This technique was tested for the first time on fish. In vivo FRI quantifies the fluorescence signals of live animals that are imaged under excitation and emission filter sets. Depth of penetration for FRI light in tissue is several centimeters, allowing for whole body imaging of small animals. The FRI analyses were conducted at Vivoptic platform (ANR-11-INBS-006, Univ. Bordeaux, CNRS, INSERM, TBM-Core, UMS 3427, US 5, F-33000 Bordeaux).

Marked (n = 8) and control live glass eels (n = 8) were anaesthetised with an eugenol solution (0.03 mL.L^-1^) before being placed one by one into a slot of an holder (a dish drainer, Sticks, Lékué, Espagne). The holder with live glass eels (4 marked, 4 control glass eels × 2) were placed into Lumina LT optical system (Perkin Elmer Inc., Boston, USA) equipped with a CDD camera (maximal field of view in the machine: 12.5 × 12.5 cm). All alive glass eels were imaged by FRI (1 s time of exposition) with two sets of excitation and emission wavelength filter: 1) 450 nm–480 nm at excitation, 515–575 nm at emission; 2) 485 nm–515 nm at excitation, 515–575 nm at emission. The imaging of glass eels was photographed (100 ms time of acquisition) and the 2D images were analysed with Living Image software (Perkin Elmer Inc., Boston, USA). The glass eels awaken were then transferred to the INRAE laboratory for the samplings.

Prior to the analyses, an alizarin drop (alizarin red S, 150 ppm, SigmaAldrich, China) was imaged (Supplementary Fig. S2b) to select the wavelengths of the excitation and emission filters. The holder was imaged with the filters selected and no autofluorescence was detected (Supplementary Fig. S2a).

### Sampling

Biometrics and fin clipping were conducted in the dark for marked ones at INRAE. Glass eels were anaesthetized with eugenol solution (0.03 mL.L^-1^) before being euthanized with eugenol solution overdose (0.3 mL. L^-1^). Total body length (± 0.01 mm), wet mass (± 0.1 mg) were measured. Pigmentation stage was determined according to^31^. A total of 85 individuals were sampled with control (n = 35, 64.82 ± 1.20 mm, 221.55 ± 18.80 mg, glass eels stage from VIA0 to VIB) and marked (n = 50, 64.81 ± 1.27 mm, 235.7 ± 17.09 mg, glass eels stage from VIA0 to VIA4) glass eels.

The tips of fins were clipped on a total of 69 glass eels (n control = 27, n marked = 42). For fluorescence spectroscopy analyses, EEM, clips of caudal fin were placed into 1.5 mL Eppendorf tubes and kept frozen (–20 °C). For epifluorescence microscopy, EPI, clips of caudal and pectoral fins were mounted between a glass slide and a cover slip. The glass slides were dried overnight at room temperature.

### Excitation-emission matrix (EEM) fluorescence spectroscopy

The analysis by EEM fluorescence spectroscopy of fish fin clips was tested for the first time. This technique provides rapidly the 3D total fluorescence spectrum of a small piece of tissue over a range of UV–visible to near IR wavelengths by creating an excitation-emission-fluorescence intensity matrix. The analyses were conducted at EPOC laboratory.

Each caudal fin clip from marked (n = 12) and control (n = 12) glass eels was prepared into a homogenate by a 20 min-sonication in 2 mL ultra-pure water (Milli-Q, Millipore, USA). Then, the homogenates were filtered (0.70 µm Whatman GF/F precombusted glass-fiber filters) and diluted to a maximum UV–vis absorbance of 0.1 (V-560 UV–VIS spectrophotometer, Jasco Corporation, Japan) to avoid inner filter effects during fluorescence analysis. The homogenates were placed into a 850 µL quartz micro-cuvette (111.057-QS, Hellma Analytics, Germany) thermo-stated at 20 °C and analysed with an Aqualog spectrofluorometer (Jobin Yvon technology, Horiba Scientific, France). The EEM fluorescence spectra (n = 24) were obtained between the wavelengths 240–800 nm at excitation (2 s integration time, 5 nm intervals) and 250–810 nm at emission (high CCD detector gain, 0.58 nm intervals). Each EEM was subtracted from the EEM spectrum of an ultrapure water blank to eliminate Rayleigh and Raman scatter peaks and corrected for instrumental biases. Prior to the analyses, the EEM spectrum of a eugenol solution (0.3 mL. L^-1^) was acquired and the solution did not fluoresce. The EEM spectrum of an alizarin solution (alizarin red S, 150 ppm, A5533, Sigma-Aldrich, China) was acquired to determine the wavelengths of the fluorescence peak of alizarin.

Based on the EEM spectra of glass eels, 15 main fluorescence emission spectra were studied in all glass eels at five fixed excitation wavelengths (Table 2). These 15 fluorescence emission spectra were acquired for each homogenate between the emission wavelengths 360–600 nm and with high resolution (0.5 s integration time, 1 nm interval) using a Fluorolog fluorometer (FL3-22 SPEX, Jobin Yvon technology, Horiba Scientific, France; 950 V) as described in^32,33^. Each fluorescence emission spectrum was subtracted from an ultrapure water blank and corrected for instrumental biases.

### Epifluorescence microscopy (EPI)

EPI analyses were conducted at INRAE. The glass slides of pectoral (n = 45) and caudal (n = 45) fin tips of marked (n = 30) and control (n = 15) glass eels (n total = 45) were photographed with an epifluorescence binocular magnifier (SMZ25, Nikon, Japan; camera: DS-Ri2, Nikon, Japan) equipped with a B-2A fluorescence filter (Nikon, excitation bandpass: 450–490 nm; dichromatic mirror cut-on: 500 nm longpass; barrier filter: 515 nm longpass). The fluorescence of each photograph was analysed using NIS-Elements BR software (version 5.02).

The fluorescence intensity observed on the photographs was assessed using a scale of 0–3 (0, no fluorescence; 1, weak autofluorescence around fin rays and at the edge of the fin as described in^15^; 2, bright and heterogeneous fluorescence of alizarin stain on fin rays and tissue; 3, very bright and homogeneous fluorescence of alizarin stain on fin tip) (Fig. 6b). Fluorescence scores were analysed independently and by blinding by three research operators. The final score was then determined by selecting the value that more than one operator had allocated to the photo. A score ≥ 2 was judged to be an acceptable and good detection of the alizarin stain.

### Statistical analyses

Statistical analyses were performed using R software (v3.6.1).

FRI provided images of the levels of radiance (photons/secondes/cm^2^/streradian) of the live glass eels. The levels of radiance between the marked and control glass eels were presented. Wilcoxon-Mann–Whitney tests (α = 0.05) were used to assess the differences in radiance between the two images obtained with the two excitation filter sets and between the marked and control groups for each image. The fluorescence quenching percentage Q (%) was evaluated with the radiance of each group for each image.

The fluorescence quenching percentage Q (%) was evaluated as follows:

$$Q \left(\%\right)=\left(1-\frac{{F}_{M}}{{F}_{C}}\right)\times 100$$

with F_M_, the fluorescence intensity of marked fish and F_C_, the fluorescence intensity of control fish. High positive values of Q (%) indicate a high fluorescence intensity quenching of the marked group compared to the control.

EEM fluorescence spectroscopy provided matrices of fluorescence intensities (in arbritary unit). The intensity at each excitation and emission wavelengths obtained by EEM were extracted to calculate within the alizarin peak (2), every 10 nm, the fluorescence intensity means, standard deviations, maximum, minimum for each group as well as the Q(%) means and standard deviation. The EEM spectra of alizarin, marked and control glass eels were described. Then, Wilcoxon-Mann–Whitney tests (α = 0.05) were used to assess the differences in the fluorescence intensities between the marked and the control group. The Q (%) means and standard deviations were evaluated with the fluorescence intensities of each group. Wilcoxon-Mann–Whitney tests (α = 0.05) were used also to assess the difference in the fluorescence intensities minima and maxima bewteen the groups in order to determine the fluorescence thresholds of each group. Patrimat analyses were conducted with the three quartiles, means, standard deviations, minima and maxima of the fluorescence intensities of each group, in order to investigate their discriminant effect. Then, quadratic nonlinear discriminant models (QDA) were assessed with the fluorescence intensities first, second and third quartiles of each glass eels group (tenfold cross-validated correctness rate, 468 observations, 3 variables, 2 classes), the most discriminant variables from Patrimat analyses. The QDA with the best correctness rate was selected and the correct classification mean percentages of each group presented.

The fluorometer provided in high resolution fluorescence peaks (in arbitrary units) whithin fluorescence emission spectra at a fixed excitation wavelength. Thus, the other approach tested to detect the alizarin signal in marked eels was to select ratios of fluorescence peaks for which their fluorescence rates was higher than for the control group. The analysis of fluorescence ratios enabled the comparison of fluorescence peaks intensities in the alizarin signal to those in the fish signal. 15 main fluorescence peaks were selected (Table 2): nine were whitin the alizarin signal (F_ARS_) and six other within the natural fish signal (F_FISH_) (Table 2). The alizarin fluorescence ratios, R_*n*_, were computed for each combination of F_ARS_ and F_FISH_ as follows:

$${R}_{{n }\;_{n:1\to 54} }=\frac{{F}_{{ARS}_{{i}\;_{ i : 1\to 9} } }}{{F}_{{FISH}_{{j }\;_{j: 1\to 6}}}}$$

with F_ARS_, the fluorescence intensity of a peak (at a fixed excitation_emission wavelength) within the alizarin signal; F_FISH_, the fluorescence intensity of a peak (at a fixed excitation_emission wavelength) within the natural signal of the fish.

When fluorescence rates of the marked group was 1% higher than for the control group, the R_*n*_ was selected. Wilcoxon-Mann-Withney test (α = 0.05) was used to evaluate the difference in the values of the selected R_*n*_ between glass eels groups. Then, QDA were run with the rates values of these R_*n*_ (tenfold cross-validated correctness rate, 38 observations, 19 variables, 2 classes) to assess whether they were adequate to assign individuals to their group. The correct classification mean percentages of each group were presented.

Epifluorescence analyses provided qualitative measures of fluorescence intensity for which a score was assigned. The variability of the assigned score amomg the three operator-researchers was evaluated. Next, Chi-square tests (α = 0.05) were used to assess differences in fluorescence scores between caudal and pectoral fins in the same group and between the marked and control groups for each fin.

### Ethics declaration

The French Ministry of the Territories and the Sea of Gironde issued authorizations to INRAE (animal experimentation approval number A33-478–001) for the capture of glass eels for biological and scientific examinations, including the killing of eels by overdosing with eugenol for finning or imaging (decree n°2018-03-13, decree n°2018-05-11). Anaesthesia and euthanasia respected the American Veterinary Medical Association (AVMA) Guidelines for the Euthanasia of Animals (2020). The captures were carried out in coordination with the Departmental Federation of Fishing of Gironde and Migado, both in charge of monitoring and managing migratory fish species, such as eels from Gironde. All methods were in strict accordance with the National Guidelines for Animal Care of the French Ministry of Agriculture (decree n°2013–118) and the EU regulations concerning the protection of animals used for scientific research (Directive 2010/63/EU). The study complied with (Animal Research) guidelines^34^. The main operator, M. G., has the diploma of fish welfare and ethics experimentation (decree 1988-04-19) to ensure direct scientific responsibility for animal experiments, delivered by ONIRIS veterinary school (France). Imaging was done at Vivoptic platform, ANR-11-INBS-006, Univ. Bordeaux, CNRS, INSERM, TBM-Core, UMS 3427, US 5, F-33000 Bordeaux, France. Vivoptic is a France Life Imaging (FLI) labelled platform (ANR-11-INBS-006).

## Supplementary Information

Supplementary Information.
