# Supplementary material for: New insights into detecting alizarin from autofluorescence in marked glass eels
Source: Sci Rep. 2022 Sep 26;12:15985. doi: 10.1038/s41598-022-18440-0 (PMC9512919; doi:10.1038/s41598-022-18440-0)
Supplement: Supplementary file 1 — Supplementary Information. [file 41598_2022_18440_MOESM1_ESM.pdf]

NEW INSIGHTS INTO DETECTING ALIZARIN FROM AUTOFLUORESCENCE IN MARKED GLASS EELS

Mélanie Gaillard[1], Edith Parlanti[2], Mahaut Sourzac[2], Franck Couillaud[3], Coralie Genevois[3], Sébastien Boutry[1], Christian Rigaud[1] and Françoise Daverat[1]

Supplementary information

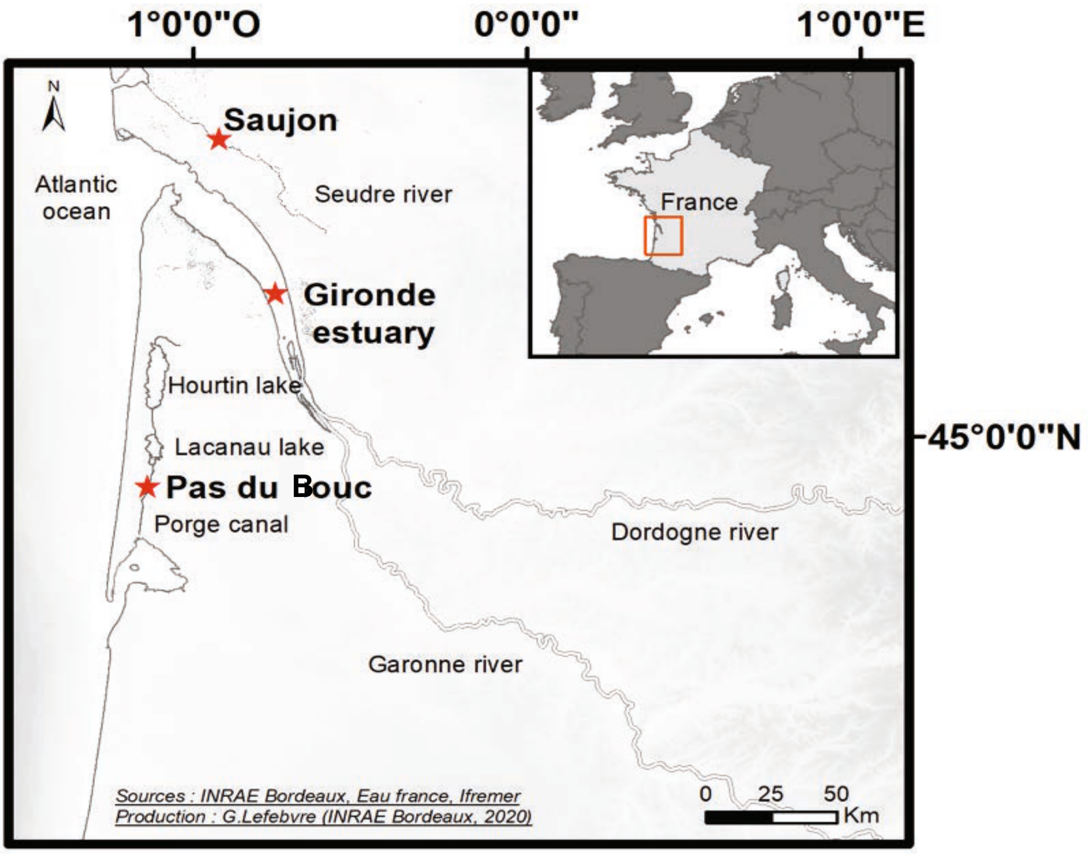

Supplementary Fig. S1. Map of the glass eels collection sites (red stars). Locations were situated in the South of France.

a

b

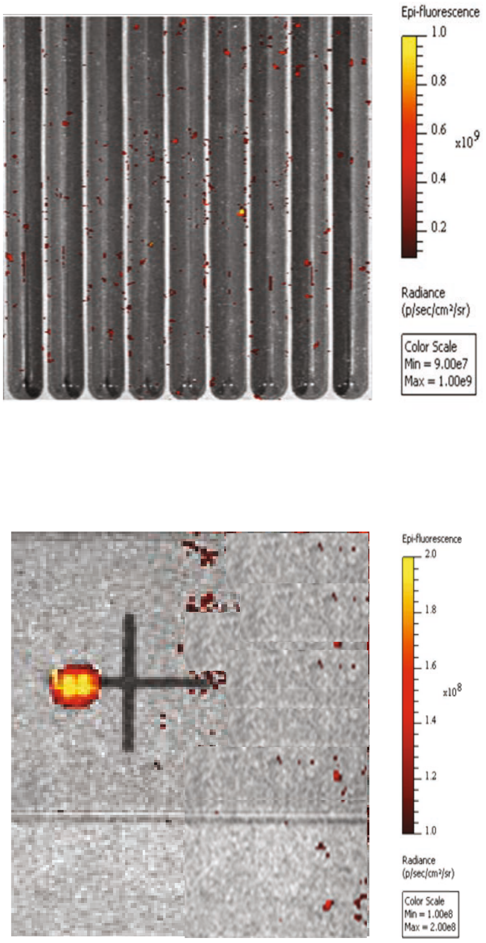

Supplementary Fig. S2. Empty holder and alizarin FRI. *In vivo* fluorescence reflectance imaging of (a) the empty holder and (b) a drop of alizarin red S (150 ppm). Fluorescence intensity was measured in p/sec/cm2/sr (photon/second/cm2/steradian).

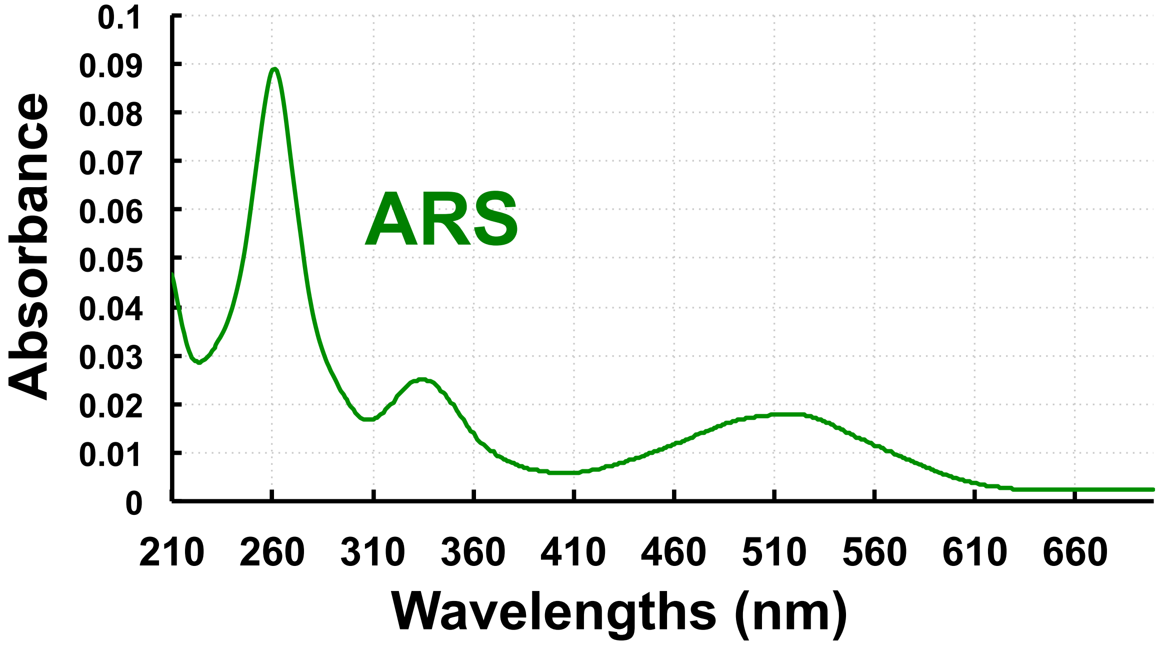

Supplementary Fig. S3. Alizarin red S, ARS, absorbance. Absorbance spectra of alizarin red S solution (150 ppm)
